# Utility of bone marrow examination in retinoblastoma and their correlation with hematological features

**DOI:** 10.25122/jml-2023-0156

**Published:** 2023-08

**Authors:** Geeta Yadav, Anurag Singh, Rashmi Kushwaha, Nishant Verma, Rajat Mohan Srivastava, Uma Shankar Singh

**Affiliations:** 1 Department of Pathology, King George’s Medical University Lucknow, India; 2 Department of Pediatrics, King George’s Medical University, Lucknow, India; 3 Department of Ophthalmology, King George’s Medical University, Lucknow, India

**Keywords:** retinoblastoma, bone marrow examination, metastasis, myelogram, bone marrow transplantation

## Abstract

Retinoblastoma makes up about 3% of all childhood malignancies. The frequency of metastatic retinoblastoma ranges from 4.8 to 11%. Assessing the bone marrow status of newly diagnosed patients is crucial because of the advantages of autologous bone marrow transplants for high-risk patients. This study aimed to determine the utility of bone marrow examination in cases of retinoblastoma and its correlation with hematological findings. This retrospective study was conducted at the Department of Pathology, King George’s Medical University, Lucknow, India. A total of 34 cases of retinoblastoma with bone marrow examination were included in the study. Bone marrow infiltration was present in 17.65% (6/34) cases of retinoblastoma. Bone marrow aspirate myelogram showed that marrow metastasis in retinoblastoma was significantly linked with a reduced percentage of total myeloid cells (p=0.001) and segmented cells (p=0.006). The present study demonstrated that 15% (3/20) of retinoblastoma patients previously classified as nonmetastatic before bone marrow examination (stages I to III based on histology, imaging, and bone scan) had bone marrow metastases following bone marrow examination and were upgraded to stage IV. To conclude, a diligent and exhaustive search for metastatic cells in bone marrow is advised if the myelogram shows a reduced percentage of total myeloid and segmented cells. All stage II and stage III cases of retinoblastoma must undergo bone marrow examination for early metastasis detection, as it may result in an upgrade to stage IV disease, impacting the prognosis and necessitating distinct treatment modalities.

## INTRODUCTION

The most common intraocular malignant neoplasm in children is retinoblastoma, with a reported frequency of 1 in 15,000 to 1 in 18,000 live births [1]. Retinoblastoma accounts for 1% of all cancer-related deaths in patients under the age of 15 years [2]. Retinoblastoma occurs in both familial and sporadic patterns. Poor vision, strabismus, a white hue, and pain with tenderness in the eye are common clinical findings at the time of presentation [3, 4]. Compared to patients without orbital invasion, the frequency of orbital invasion is linked to a 10- to 27-fold higher risk of metastasis [5, 6]. Optic nerve invasion with or without the involvement of a resection margin, choroidal invasion, and enucleation of an affected eye more than 120 days after initial diagnosis have all been demonstrated to be independently linked with the emergence of metastases [7]. Metastasis occurs in approximately 4.8% to 11% of retinoblastoma cases [8, 9], with the bone marrow (BM) being the most common site for metastatic spread. Accurate identification of bone marrow involvement is critical for determining the stage of the tumor. The prognosis, therapeutic response, and treatment regimens are all influenced by bone marrow metastases [10]. It is important to assess the bone marrow status of newly diagnosed children since the effectiveness of autologous bone marrow rescue is being explored for patients with metastatic retinoblastoma. Bone marrow examination is frequently used for this purpose, primarily because it is a rapid, straightforward, and cost-effective procedure. While the evaluation of bone marrow metastasis by solid tumors has been the focus of extensive research in the past [11, 12], only a limited number of studies have investigated the importance of BM examination in retinoblastoma and its correlation with hematological findings. There are currently no accepted guidelines describing when a bone marrow examination should be performed in relation to the various stages of retinoblastoma. The aim of this study was to determine the usefulness of BM examination in cases of retinoblastoma and its correlation with hematological findings.

## MATERIAL AND METHODS

This retrospective study was conducted in the Department of Pathology in collaboration with the Department of Pediatrics at King George’s Medical University, Lucknow, India. All cases of retinoblastoma in which BM examinations were conducted from January 2017 to May 2022 were included in the study. Inclusion criteria for this study encompassed individuals of any age and gender diagnosed with retinoblastoma and who had undergone a comprehensive assessment, including a complete blood count, bone marrow examination, and clinical and radiological examinations. The BM procedure was performed from the unilateral posterior superior iliac spine (PSIS) after the informed consent of their close relatives. Subsequently, BM aspirate smears and biopsy sections were prepared and stained using Leishman stain and hematoxylin and eosin (H-E stain), respectively, per standard protocols [13, 14]. A detailed chart review was done from bone marrow case sheet records and the hospital information system (HIS). In all cases, demographic details, clinical-radiological findings, a complete blood count, and morphological findings of peripheral blood and bone marrow aspirate smears were extracted from the available records. Cases of retinoblastoma with incomplete evaluation and/or missing peripheral blood and BM examination findings were excluded from the study. Patients on chemotherapy or radiotherapy were also excluded from the study.

All retinoblastoma patients admitted to our institution had baseline examinations, including orbital ultrasonography, contrast-enhanced computed tomography (CECT) scan or magnetic resonance imaging (MRI) of the orbit and head, and clinical evaluation of the eyes under local anesthesia. These initial radiographic scans of the head and orbit allowed for the detection of overt orbital disease, such as extraocular mass, thickening of the optic nerve, or central nervous system (CNS) metastases.

In cases of retinoblastoma, reports of histopathology were evaluated for high-risk factors, such as tumor cell invasion in the sclera, iris, choroid, anterior chamber, iris, or optic nerve (retrolaminar invasion without the involvement of its resected margin), and residual microscopic disease, such as involvement of the resection end of the optic nerve or extra-scleral tissue by tumors cells, in patients who had enucleation. The International Retinoblastoma Staging System (IRSS) classification was used to classify all cases. In each case included in the study, a BM examination (unilateral BM aspirate and biopsy) was performed. The presence of metastatic round cell tumors on aspirate and/or biopsy sections, along with the expression of synaptophysin and neuron-specific enolase (NSE) immunohistochemistry, was used to determine BM positivity. The present study included 34 retinoblastoma cases with bone marrow examination.

### Statical analysis

Demographic, clinical, and hematological features of the cases were summarized using mean and standard deviation. Associations between categorical variables were analyzed using the Chi-square test and Fisher’s exact test. Variables that demonstrated a significant association with the outcome were reported as p<0.05.

## RESULTS

Peripheral blood and bone marrow examinations were evaluated in 34 cases of retinoblastoma in the current study. The median age at presentation was 4.0 years. The disease was bilateral in 14.71% (5/34) and unilateral in 85.29% (29/34) cases. The male-female ratio was 1.6:1 among the study population. White reflex (61.76%) and squint (11.76%) were the two first symptoms that were most frequently reported. The bone marrow metastasis was noted in 17.65% (6/34) cases of retinoblastoma. The mean hemoglobin (gm/dl), total leucocyte count (x10^9^/L), and platelet count (x10^9^/L) were 10.87±1.96, 12.65±4.48 and 4.07±1.31 respectively, in retinoblastoma cases. There was no significant difference noted for gender (p=0.785), mean age (p=0.347), hemoglobin (Hb) (p=0.605), total leucocyte count (TLC) (p=0.702), and platelet count (PC) (p=0.253) in cases of retinoblastoma with and without bone marrow metastasis. None of the cases of bilateral retinoblastoma showed bone marrow metastasis in the present study (Table 1).

**Table 1:** Demographic profile and hematological parameters in cases of retinoblastoma

Parameters		Total number of retinoblastoma cases (n=34)	Retinoblastoma with bone marrow infiltration (n=6)	Retinoblastoma without bone marrow infiltration (n=28)	p-value
Age (years)	Mean ± Standard Deviation	4.12±2.49	5.00±2.54	3.93±1.41	0.347
Gender (n,%)	Male	21 (61.76%)	4 (66.67%)	17 (60.71%)	0.785
	Female	13 (38.24%)	2 (33.33%)	11 (39.29%)	
Unilateral/ Bilateral (n,%)	Bilateral	5 (14.7%)	0 (0.0%)	5 (100%)	<0.001
	Unilateral	29 (85.3%)	6 (20.7%)	23 (79.3%)	
Hemoglobin (gm/dl)	Mean ± Standard Deviation	10.87±1.96	10.48±2.38	10.95±1.90	0.605
Total leukocyte count (x10^9^/L)	Mean ± Standard Deviation	12.65±4.48	13.30±3.33	12.51±4.73	0.702
Platelet count (x10^9^/L)	Mean ± Standard Deviation	4.07±1.31	4.63±1.02	3.95±1.34	0.253

### Hematological findings in retinoblastoma cases with and without bone marrow metastasis

The complete blood count revealed anemia in 73.53% (25/34), leucocytosis in 55.88% (19/34), and thrombocytosis in 35.29% (12/34) of cases with retinoblastoma. There was no significant correlation seen for anemia (p=0.928), leucocytosis (p=0.894), and thrombocytosis (p=0.193) in cases of retinoblastoma with and without bone marrow infiltration. The cut-off values for anemia, leucocytosis, and thrombocytosis were 11.0g/dL, 11.0x10^9^/L, and 450x10^9^/L, respectively [14].

It was observed that bone marrow metastasis was also not significantly linked with the presence of nucleated red blood cells (p=0.248) and shift cells (p=0.433) in peripheral blood (Table 2).

**Table 2:** Evaluation of peripheral hematological parameters in retinoblastoma cases with and without marrow metastasis

Parameters	Total number of retinoblastoma cases (n=34)		Retinoblastoma with bone marrow infiltration (n=06)		Retinoblastoma without bone marrow infiltration (n=28)		p-value
	n	%	n	%	n	%	
Anemia	25	73.53	5	83.33	20	71.43	0.928
Leucocytosis	19	55.88	4	66.67	15	53.57	0.894
Thrombocytosis	12	35.29	4	66.67	8	28.57	0.193
Nucleated Red Blood Cells	8	23.53	3	50.00	5	17.86	0.248
Shift Cell	5	14.71	2	33.33	3	10.71	0.433

### Myelogram characteristics in cases of retinoblastoma with and without bone marrow metastasis

**Figure 1:**
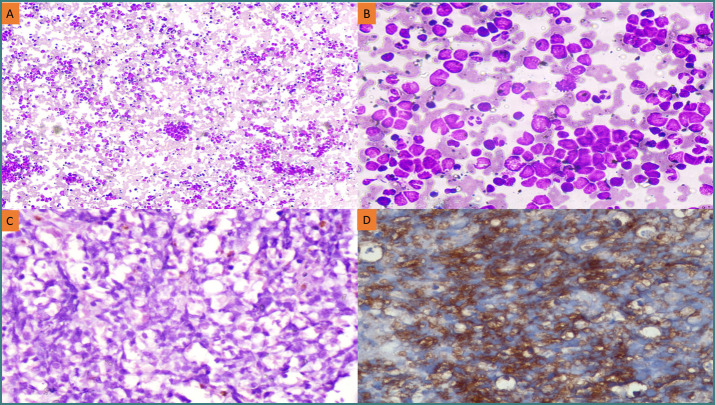
Bone marrow examination and immunohistochemistry findings in retinoblastoma. (A) Bone marrow aspirate smear displaying metastasis by retinoblastoma (Leishman stain, 100x); (B) Bone marrow aspirate smear displaying infiltration by tumor cells disposed in the form of rosettes (Leishman stain, 400x); (C) Section of bone marrow biopsy showing metastasis by retinoblastoma (hematoxylin and eosin stain, 400x); (D) Immunohistochemical stain on bone marrow biopsy showing expression of synaptophysin in tumor cells, 400x.

Bone marrow aspirate smears and trephine biopsy findings were retrieved in each case of retinoblastoma. In six of the 34 cases, aspirate smears and trephine biopsy revealed metastasis with the expression of synaptophysin and neuron-specific enolase (NSE) (Figure 1 A-D).

The bone marrow aspirate myelogram showed that marrow metastasis in retinoblastoma was significantly linked with a reduced percentage of total myeloid cells (p=0.001) and segmented cells (Bands and neutrophils) (p=0.006). The other parameters in the myelogram did not show a significant correlation (Table 3).

**Table 3:** Comparison of bone marrow aspirate smear morphological parameters in retinoblastoma cases with and without marrow metastasis

Parameters (%)	Total number of Retinoblastoma cases (n=34)	Retinoblastoma with bone marrow infiltration (n=06)	Retinoblastoma without bone marrow infiltration (n=28)	p-value
	Mean ± SD	Mean ± SD	Mean ± SD	
Total Myeloid cell	56.03±15.40	38.33±11.98	59.82±13.38	0.001
Promyelocytes	1.97±0.63	2.17±0.41	1.93±0.66	0.407
Myelocytes	11.68±6.15	10.33±6.31	11.96±6.19	0.563
Metamyelocytes	8.35±5.15	6.17±3.43	8.82±5.38	0.258
Band and neutrophils	33.76±14.44	19.67±8.82	36.79±13.67	0.006
Total Erythroid cells	25.88±12.25	18.00±10.73	27.57±12.05	0.082
Lymphocytes	11.53±7.56	12.83±8.04	11.25±7.58	0.649
Plasma cells	1.85±0.78	2.33±1.03	1.75±0.70	0.099

p-value: <0.05 (Significant correlation)

The most prevalent pattern of metastasis in the biopsy section was rosette formation, which was observed in 66.66% (4/6), followed by diffuse infiltration in 16.66% (1/6) and focal interstitial pattern in 16.66% (1/6) of trephine biopsies. The mixed pattern of metastasis was noted in 66.66% (4/6) of cases.

### Retinoblastoma staging

#### Staging of retinoblastoma cases before bone marrow examination

Of the 34 cases with retinoblastoma, 8.82% (3/34) were stage I, 17.65% (6/34) were stage II, 32.35% (11/34) were stage III, and 41.18% (14/34) were stage IV based on clinical, radiological (CT/MRI orbit and head, whole body PET scan), and histomorphology findings of an enucleated eye.

#### Staging of retinoblastoma cases after bone marrow examination

Among the 8.82% (3/34) cases in stage I, none of the patients showed BM metastasis. Within the 17.65% (6/34) cases initially classified as stage II, 16.66% (1/6) showed BM metastasis, resulting in an upgrade to stage IV. Of the 32.35% (11/34) cases initially classified as stage III, 18.18% (2/11) showed BM metastasis leading to an upgrade to stage IV. After bone marrow examination, 15% (3/20) of cases in stages I, II, and III were upgraded to stage IV. Among stage IV retinoblastoma patients, 21.43% (3/14) showed BM metastasis. Three cases were upgraded from stages II and III after BM examination, leading to 17.65% (6/34) of cases positive for bone marrow metastasis with retinoblastoma (Table 4).

**Table 4:** Percentage of bone marrow involvement in different stages of retinoblastoma

Retinoblastoma Stage	Total number of Retinoblastoma cases before bone marrow examination (n=34)	Total number of Retinoblastoma cases after bone marrow examination (n=34)
I	8.82% (3/34)	8.82% (3/34)
II	17.65% (6/34)	14.71% (5/34)
III	32.35% (11/34)	26.47% (9/34)
IV	41.18% (14/34)	50% (17/34)

The treatment regimen for retinoblastoma cases included a combination chemotherapy using the drugs carboplatin, etoposide phosphate, and vincristine sulfate, along with the application of radiotherapy. In addition, among cases of retinoblastoma with bone marrow metastasis, high-dose chemotherapy was used, followed by referral for stem cell rescue.

## DISCUSSION

Retinoblastoma accounts for 3% of pediatric malignancies [15]. In patients with retinoblastoma, the bone marrow, followed by the bones and liver, are the most frequent sites of metastasis [16]. Few studies have been done to examine the infiltration paradigm in retinoblastoma. A study by Muhammad *et al*. showed that 25% (20/80) of patients with retinoblastoma showed BM metastasis at initial presentation [17]. Selistre *et al*. reported that 10.7% (15/140) of retinoblastoma patients in Brazil had bone marrow metastases [18]. Hu H *et al*. found that 4.16% (1/24) of cases showed bone marrow metastasis [19]. According to the findings of our study, 17.65% (6/34) of the patients had bone marrow metastases. The varying sample sizes, stages at initial presentation, and geographic diversity may all contribute to the heterogeneity in bone marrow metastasis results.

The type of treatment chosen for retinoblastoma patients depends upon the stage of the disease, and the patient's prognosis is determined by whether the primary tumor has metastasized or not [20]. Massive choroidal invasion, anterior segment seeding, iris, and ciliary body infiltration are risk factors for retinoblastoma metastasis. Brain and orbit magnetic resonance imaging (MRI) with and without contrast, abdomen computed tomography (CT), bone scan, and bone marrow examination are all part of the systemic workup for suspected metastatic disease [21]. In situations where post-enucleation histomorphology reveals the presence of high-risk factors such as tumor infiltration into extra-scleral tissue or invasion of the resection end of the optic nerve, a BM examination should be performed to stage the disease accurately. A bone marrow examination is required if there is an orbital extension, regional lymph node involvement, symptoms of the central nervous system (CNS), or systemic spread [22]. These studies show the importance and justification of bone marrow workup for most affected children with retinoblastoma. The Children's Oncology Group is running a trial to treat bone marrow metastatic retinoblastoma (COG ARET 0321: A Trial of Intensive Multimodality Therapy for Extraocular Disease Protocol), which entails high-dose chemotherapy with etoposide, carboplatin, and thiotepa followed by stem cell rescue. Depending on the response, involved field external beam radiotherapy may subsequently be considered [23-25]. Inspiring new research suggests that individuals with distant metastatic retinoblastoma without central nervous involvement who receive high-dose chemotherapy combined with autologous stem cell rescue have better survival [10, 26, 27]. The survival rate for metastatic retinoblastoma is often less than 10%, and treatment is challenging. Early metastatic detection may aid in high-dose chemotherapy and stem cell transplantation, which could improve these patients' likelihood of surviving. Patients with retinoblastoma diagnosed without overt metastases get BM evaluations in light of this risk [28].

In our study, anemia was noted in 73.53% (25/34) cases of retinoblastoma. This could be explained by the participation of the retinoblastoma protein-interacting zinc finger protein (RIZ) or positive regulatory domain methyl transferase (PRDM2), which has tumor-suppressive capabilities, in the process of erythropoiesis, which may also involve the RB gene. There is a loss of heterozygosity or deletion of this gene in a number of cancers, including hematological malignancies [29]. In the present study, there was no significant correlation for hemoglobin, leucocytosis, platelet count, presence of nucleated red blood cells, or shift cells in cases of retinoblastoma with and without bone marrow infiltration.

The bone marrow aspirate myelogram showed that marrow metastasis in retinoblastoma was significantly linked with a reduced percentage of total myeloid cells and segmented cells (bands and neutrophils). According to a prior study, when tumor cells metastasize to the bone marrow, a large number of tumor cell proliferation may affect the hematopoietic microenvironment, inhibiting normal hematopoietic cell growth. However, if the invasion of a small number of tumor cells occurs, the hematopoietic microenvironment may be normal or hyperactive [30]. There are no such studies that have analyzed the results of myelograms in patients with retinoblastoma; hence, we cannot compare our findings.

In this study, 15% (3/20) of patients with retinoblastoma thought to be nonmetastatic (stages I to III based on histology, imaging, and bone scan) revealed marrow metastases on BM examination and were upgraded to stage IV disease. The literature review showed a range of 0% to 1.7% for bone marrow positivity in similar cases [31-34]. The higher percentage in our study may be explained by the smaller sample size and the exclusion of stage 0 and stage I cases without high-risk features for BM metastasis. In the present study, 16.66% (1/6) cases classified as stage II and 18.18% (2/11) cases classified as stage III retinoblastoma showed BM metastasis. A few previous studies have reported 6% and 11% positivity for bone marrow in stage II and III retinoblastoma patients [34]. We did not identify BM metastases in stage I disease, in conformity with previous studies. Few authors have advocated BM examination in stage I retinoblastoma cases with high-risk features despite the lack of evidence to support this [31-34, 35]. In stage II and III patients, bone marrow examination must be done to rule out microscopic metastasis, as the present study showed bone marrow metastasis at presentation in 16.66% (1/6) of stage II patients and 18.18% (2/11) of stage III patients. Early detection of metastasis in such cases will lead to changes in treatment modalities and the prognosis of retinoblastoma.

## CONCLUSION

To conclude, a diligent and exhaustive search for metastatic cells in bone marrow is advised if the myelogram shows a reduced percentage of total myeloid and segmented cells. All stage II and stage III cases of retinoblastoma must undergo BM examination for early metastasis detection, as it may result in an upgrade to stage IV disease, impacting the prognosis and necessitating distinct treatment modalities such as stem cell transplantation and high-dose chemotherapy.
